# Reward can modulate attentional capture, independent of top-down set

**DOI:** 10.3758/s13414-015-0958-6

**Published:** 2015-07-16

**Authors:** Jaap Munneke, Sylco S. Hoppenbrouwers, Jan Theeuwes

**Affiliations:** Department of Cognitive Psychology, Vrije Universiteit, Amsterdam, The Netherlands

**Keywords:** Selective attention, Attentional capture

## Abstract

The traditional distinction between exogenous and endogenous attentional control has recently been enriched with an additional mode of control, termed “selection history.” Recent findings have indicated, for instance, that previously rewarded or punished stimuli capture more attention than their physical attributes would predict. As such, the value that is associated with certain stimuli modulates attentional capture. This particular influence has also been shown for endogenous attention. Although recent leads have emerged, elucidating the influences of reward on exogenous and endogenous attention, it remains unclear to what extent exogenous attention is modulated by reward when endogenous attention is already deployed. We used a Posner cueing task in which exogenous and endogenous cues were presented to guide attention. Crucially, the exogenous cue also indicated the reward value. That is, the color of the exogenous cue indicated how much reward could be obtained on a given trial. The results showed main effects of endogenous *and* exogenous attention (i.e., speeded reaction times when either cue was valid, as compared to when it was invalid). Crucially, an interaction between exogenous cue validity and reward level was observed, indicating that reward-based associative-learning processes rapidly influence attentional capture, even when endogenous attention has been actively deployed.

Traditionally, a distinction has been made between endogenous and exogenous visual selective attention, in which “endogenous” or “top-down” attention refers to controlled or goal-driven attentional guidance, whereas “exogenous” or “bottom-up” attention refers to stimulus-driven guidance of attention. The latter form of attentional guidance is primarily driven by the physical features of external stimuli, such as their relative salience (Posner, Snyder, & Davidson, 1980; Wolfe, 1994; Wolfe, Cave, & Franzel, 1989). The phenomenon in which attention is reflexively guided to certain stimuli is often referred to as “attentional capture”, emphasizing its involuntary and automatic nature (e.g., Theeuwes, 1992, 2010).

However, the classic dichotomy of attentional guidance has recently been called into question and deemed incomplete, because converging evidence has shown patterns of attentional allocation not fitting either of these classic models. For example, it has recently been shown that previously rewarded (Anderson, Laurent, & Yantis, 2011; Failing & Theeuwes, 2014) or punished (Schmidt, Belopolsky, & Theeuwes, 2015) stimuli capture attention to a larger extent than would be expected on the basis of their physical features or the observer’s current task-induced goals. In addition, the phenomenon of contingent capture (i.e., the idea that attentional capture depends on the individual’s top-down set; Folk & Remington, 2006; Folk, Remington, & Johnston, 1992) can be explained in terms of selection history, instead of a combination of top-down and bottom-up factors (Belopolsky, Schreij, & Theeuwes, 2010).

As such, the endogenous–exogenous dichotomy cannot explain why certain stimuli draw increased visual attention independent of their physical salience or the current goals of the observer. Recently, evidence has accumulated that suggests a separate class of attentional processes. An alternative framework has been described in which “selection history” is put forth as a third category (Awh, Belopolsky, & Theeuwes, 2012). More precisely, Awh and colleagues stated that the attentional priority map (Wolfe et al., 1989) should be extended beyond integration of the current goals of the observer and the physical salience of stimuli, and should include selection history as a third means of attentional control. *Selection history*, in this context, refers to a lingering effect of past selection criteria that modulates the current attentional deployment. Thus, attention can be guided by selection criteria that were previously used to successfully select specific target stimuli, even though in the current task they are no longer relevant.

Reward history influences attentional deployment, such that attention is drawn to (features of) stimuli that have previously been associated with the attainment of reward (Bucker & Theeuwes, 2014; Failing & Theeuwes, 2014; Stankevich & Geng, 2014). Thus, involuntary shifts of attention may occur solely on the basis of the presence of previously rewarded stimuli. The influence of prior reward is clearly neither endogenous nor exogenous in the classical sense, but exerts its influence on both forms of attentional guidance.

The manner in which reward history influences attentional guidance has been explored in recent years. For example, Della Libera and Chelazzi (2006) showed a strong influence of reward on endogenous attentional guidance by employing a priming paradigm in which participants had to attend to and select local or global features from both a prime and a subsequent probe. Critically, the selected features of the prime and probe could be congruent (similar feature) or incongruent (different feature). In their particular task, the prime was associated with either a high or a low monetary reward. The results of this priming study showed a consistent negative priming effect after a high-reward prime, but this effect was absent after low-reward primes. These findings demonstrate that reward attaches value to stimuli, or to features of stimuli, by reinforcing the contingencies that lead to attentional selection.

With regard to attentional capture, the paradigm used by Anderson and colleagues (Anderson, 2013; Anderson, Laurent, & Yantis, 2011) shows that salient stimuli can become more salient when associative-learning processes attach a reward value to such stimuli. In this value-driven attentional capture paradigm, participants respond to the orientation of a target line presented within a shape singleton target (e.g., a colored diamond, surrounded by colored distractor circles). The crucial finding in of this experiment is that reaction times (RTs) are slowed if one of the distractor circles is presented in a color that was rewarded in an earlier, independent training session. The claim is that attention was captured by the distractor circle presented in the rewarded color, leading to slowed selection of the target stimulus. However, in this paradigm one possible pitfall is that the feature that is related to a certain reward outcome (i.e., color) is concurrently selected with the target. As such, increased attentional capture after a training phase may not necessarily reflect the value of a feature, but may be associated with repeatedly selecting that feature—an argument that has been advanced by Le Pelley, Pearson, Griffiths, and Beesley (2015). In the adaptation of the value-driven attentional capture paradigm by Le Pelley and colleagues, it is the *distractor* that predicts whether reward can be obtained, but it is the response to the *target* that determines whether reward is actually obtained. Therefore, the authors provided evidence that value-driven attentional capture is indeed driven by associative learning about reward contingencies, rather than by the repeated selection of an incidentally concurrent feature.

The study by Le Pelley and colleagues (2015) clearly showed value-driven attentional guidance to the location of the rewarded distractor, prior to selecting the correct target location. A question that remains unanswered is to what extent value-driven attentional capture occurs, once the most likely location of the target is selected in advance. More specifically, to what extent does a rewarded salient distractor capture attention after endogenous attention has been deployed? To answer this question, we used a Posner cueing task in which both endogenous and exogenous cues were used. Crucially, the exogenous cue signaled differential reward value and was displayed after the presentation of the endogenous cue. We hypothesized that exogenous cues indicating higher reward would capture more attention, reflected in speeded RTs when the exogenous cue was valid, and slowing of RTs when the exogenous cue was invalid.

On the basis of prior findings, the outcome of this experiment could be hypothesized to go in two opposite directions. On the one hand, it has been shown that attentional capture by salient stimuli is strongly diminished when attention has been deployed to a precued target location (Grubb, White, Heeger, & Carrasco, 2015; Theeuwes, 1991). These findings were corroborated by a different line of research, in which it was shown that attentional capture by salient distractors is strongly diminished when the distractors are presented outside the attentional window (Belopolsky & Theeuwes, 2010; Belopolsky, Zwaan, Theeuwes, & Kramer, 2007).

On the other hand, prior work also using a Posner cueing task showed that exogenous and endogenous attention can operate simultaneously and independently of each other (Berger & Henik, 2000; Chica, Botta, Lupiáñez, & Bartolomeo, 2012). These findings suggest that despite prior attentional allocation, exogenous cues can still influence attentional guidance. Therefore, it is possible that value-driven attentional capture still takes place, despite the deployment of endogenous attention. However, since none of these studies have investigated the effect of value-driven attentional capture on endogenous attentional deployment, it is unclear whether these two means of attentional guidance operate independently of each other, or whether they interact in the context of reward.

## Method

### Participants

We tested 18 healthy participants (11 females, seven males; mean age ± standard deviation: 23.8 ± 2.8 years) with normal or corrected-to-normal vision. All gave written informed consent and were paid for participation. The procedures were approved by the local ethical committee, and in accordance with the Declaration of Helsinki (“WMA Declaration of Helsinki—Ethical Principles for Medical Research Involving Human Subjects,” 2013).

### Stimuli and procedure

In this within-subjects design, participants took part in a reward version and a nonreward (control) version of the experiment on two separate days, with session order (reward/control) counterbalanced over participants. Stimulus presentation was controlled using Psychophysics Toolbox 3.0.11 (Brainard, 1997; Pelli, 1997) for MATLAB (MathWorks, Inc), running on an HP Compaq 6300 Pro computer and presented on a 120-Hz Samsung Syncmaster 2233, with a screen diagonal of 22 in. Participants were seated in a dimly lit room, at a distance of 75 cm from the monitor. Viewing distance was kept constant using a chinrest.

#### Reward cueing task

**Fig. 1:**
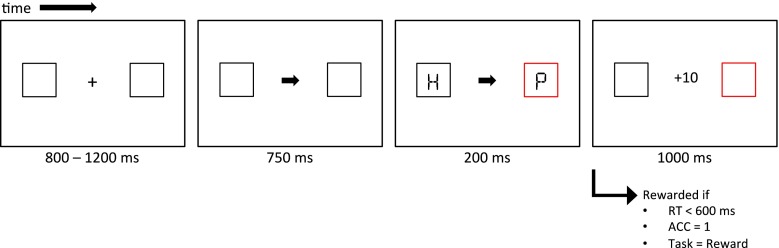
Time course of a typical reward trial

Figure 1 shows the time course of a typical reward trial. All stimuli were presented on a light gray background (28.0 cd/m^2^). Participants were instructed not to make eye movements during a trial and to focus on a small gray fixation cross, presented in the center of the display (0.23° × 0.23° of visual angle). Throughout a trial, two gray square placeholder boxes (2.14° × 2.14°) flanked the fixation cross to the left and the right at a distance of 5.72 visual degrees. After a random interval between 800 and 1,200 ms (100-ms increments), a centrally presented endogenous cue appeared in the form of a small gray arrow pointing left or right (0.53° × 0.46°), which would stay on the screen until the end of the trial. The endogenous cue (arrow) had a predictive validity of 80 %, and participants were explicitly informed of the high validity of this cue. After 750 ms, one of the two boxes would change to one of three colors, whereas the other box would remain gray. At the same time, a letter (0.61° × 1.15°) was presented in each box. One of the two letters was always the target letter “P” or “S,” whereas the letter in the box not containing the target always consisted of a randomly selected distractor letter “E” or “H.” The exogenous cue (the colored box) contained the target on exactly 50 % of the trials. Participants were explicitly informed that the colored box did not predict the target’s location. Participants were instructed to respond to the identity of the target letter as quickly as possible, using the letters “z” and “m” on a standard keyboard. The letters stayed on the screen for 200 ms, after which participants were given an additional 1,300 ms to respond. Immediately after a response was given or when 1,500 ms since target onset had passed, the reward screen appeared, informing participants of how many points they had obtained on this particular trial.

The three colors of the exogenous cue were associated with three reward levels: no reward (0 points), low reward (2 points), and high reward (10 points). The used colors were selected from a subset of six near-equiluminant colors (red, green, blue, yellow, purple, and turquoise) with an approximate luminance of 28.6 cd/m^2^ (*SD* = 2.5 cd/m^2^). Reward colors and contingencies were counterbalanced over participants. Reward was only provided when participants responded accurately and within 600 ms after target onset. If participants gave an accurate response that was slower than 600 ms (but within 1,500 ms after target onset), they would obtain 0 points. If participants did not respond within 1,500 ms after target onset, or responded incorrectly, they would lose 5 points. The number of points earned on a trial would be presented at the end of the trial for a duration of 1,000 ms.

At the beginning of the experiment, participants were informed that reward administration was determined by their performance as well as by the stimuli presented on the screen. No direct association between a color and a reward was provided in the instructions. At the end of the experiment, obtained points were converted to money, such that participants could maximally obtain an additional €8 in addition to the hourly fee (€8/h).

The reward cueing task consisted of seven blocks, each containing 120 trials, for a total of 840 trials in the experiment. A short practice block consisting of 20 trials preceded the experiment. One session took approximately 70 min to complete.

#### Control task

In addition to the reward-cueing task, the same participants performed a nonrewarded control task. Using highly similar reward and nonreward (control) tasks in a within-subjects design can provide additional insight into the relationship between endogenous and exogenous attentional guidance in the presence or absence of reward learning. Specifically, the control task was included to address two independent questions. First, to what extent does the association of reward value with color lead to value-driven attentional capture, and to what extent is this effect driven by the relative physical salience (i.e., by the exogenous cue being the only colored item in the display)? In other words, this control task was important to show that the task manipulation was successful, in that both endogenous and exogenous cues guided attention. Second, to what extent can one compare the no-reward trials in the control task with the no-reward trials in the reward task? Although these trials are physically similar, it might be the case that exogenous attention by physical salience is diminished in the reward-cueing task because of added attentional control settings due to reward.

Participants performed a task that was highly similar to the reward-cueing task, with two major differences. First, three new colors were selected from the subset of equiluminant colors and were used for the exogenous cues. These new colors were chosen such that no carryover effects from the reward task to the control task could take place. Second, no reward was administrated in the task. Instead of reward feedback, participants were given feedback with the words “correct,” “incorrect,” or “too slow,” using the same criteria as in the reward-cueing task. Three distinct colors were used to guide exogenous attention, to make the control task look similar to the reward task, even though the three colors were not associated with any value. Other than these differences, the control task was similar in all respects to the reward-cueing task.

It is important to notice that by using an endogenous symbolic cue and a salient exogenous cue, we expected to observe classic bottom-up and top-down effects of attention. Crucially, we expected that the reward manipulation would lead to additional attentional capture that was neither bottom-up nor top-down, but purely value-driven, as a direct result of the learned stimulus–reward associations.

## Results

### Reward-cueing task

#### Reaction times

In order to gain a better understanding of how different sources of attentional guidance may interact, a repeated measures analysis of variance (ANOVA) was performed on the RTs of the rewarded cueing task (see Table 1 for all mean RTs and accuracy scores). The RTs included in the analysis were derived from trials in which the participants responded correctly (8.9 % discarded) with an RT between 200 and 1,000 ms (less than 1 % of data discarded). This means that trials were included in which no reward was obtained (RTs between 600 ms and 1,000 ms). The logic behind this was twofold: (1) Participants only knew whether they would obtain a reward at the end of the trial, suggesting that the motivation after the reward cutoff of 600 ms did not change. (2) The influence of prior reward on the RTs was independent of the cutoff used on a single trial, but depended on the learned associations between color and reward in previous trials. The within-subjects factors were Reward Level (high, low, none), Exogenous Cue Validity (valid, invalid), and Endogenous Cue Validity (valid, invalid). As expected, a main effect of endogenous cue validity was observed, showing that participants responded faster to a cue that indicated the correct location of the target (valid = 422 ms, invalid = 453 ms) [*F*(1, 17) = 10.604, *p* = *.*005, *η*_p_^2^ = .384, power = .866]. Additionally, a main effect of exogenous cue validity was observed, showing that participants were faster to respond to the target when the target was presented in the colored box (valid = 432 ms, invalid = 443 ms) [*F*(1, 17) = 13.985, *p* = *.*002, *η*_p_^2^ = .451, power = .941]. Although no main effect of reward was observed (*F* < 1), we did find a significant interaction between reward level and exogenous cue validity [*F*(2, 34) = 7.305, *p* = *.*008, *η*_p_^2^ = .301, power = .814, Greenhouse–Geisser corrected]. This interaction indicates that reward influenced the extent to which attention was captured by the reward-associated stimuli. By contrast, endogenous cue validity did not interact with reward level or exogenous cue validity (*F*s < 1). No three-way interaction between the two cue types and reward level was observed (*F* < 1).

**Table 1:** Mean reaction times in milliseconds (RT) and percentages of correct responses (Acc) per condition

		Endogenous Validity			
		Valid		Invalid	
	Exogenous Validity	RT	Acc	RT	Acc
Reward Task					
High reward	Valid	410 (6)	92.8 (1.2)	441 (12)	87.7 (2.3)
	Invalid	434 (8)	91.2 (1.5)	463 (10)	87.7 (2.3)
Low reward	Valid	418 (8)	92.9 (1.2)	447 (11)	90.3 (2.1)
	Invalid	425 (7)	92.9 (1.2)	456 (11)	89.2 (1.9)
No reward	Valid	421 (9)	92.5 (1.4)	455 (12)	90.2 (1.9)
	Invalid	425 (9)	93.5 (0.9)	455 (11)	87.8 (1.7)
Control Task					
No reward	Valid	427 (8)	91.9 (1.0)	458 (11)	86.8 (2.2)
	Invalid	433 (9)	92.3 (0.9)	468 (12)	85.6 (2.4)

The values in brackets represent the standard errors of the means.

**Fig. 2:**
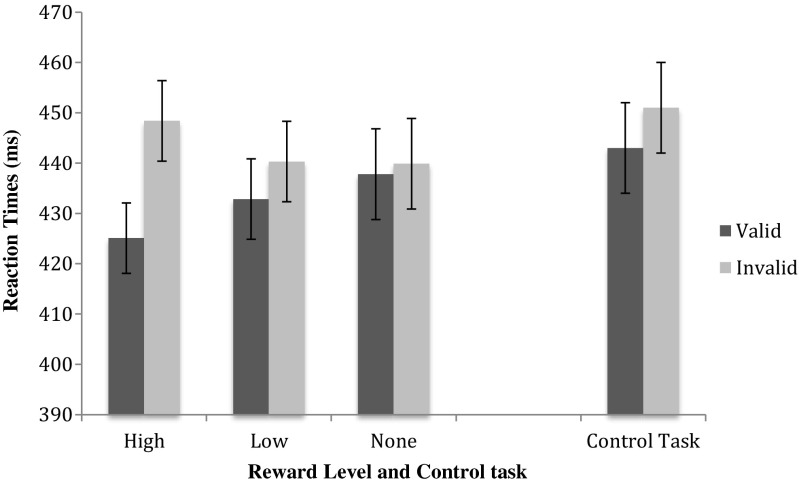
The exogenous validity effect per reward level increases with higher rewards. Error bars reflect the standard errors of the means. The effects of the reward-cueing task are contrasted with the control task

To investigate how reward associations influenced exogenous attention, we examined the exogenous cue validity effects separately for each reward level. A repeated measures ANOVA with Endogenous and Exogenous Cue Validity as within-subjects factors was performed for each reward level separately (see Table 1 for the average RTs and accuracy scores per condition). This analysis showed a significant exogenous validity effect for high-reward trials [*F*(1, 17) = 18.484, *p* < .001, *η*_p_^2^ = .521, power = .982] and a trend for low-reward trials [*F*(1, 17) = 3.341, *p* = *.*085, *η*_p_^2^ = .164, power = .407], whereas no reliable difference was observed for the no-reward trials (*F* < 1; see Fig. 2).

In addition, a linear trend was observed between the magnitude of the exogenous cue validity effect and reward level, showing that higher reward levels were associated with stronger attentional capture effects [*F*(1, 17) = 11.515, *p* = *.*003, *η*_p_^2^ = .404, power = .892]. A separate analysis showed marginal linear trends for both valid and invalid exogenous cues separately [valid: *F*(1, 17) = 4.094, *p* = *.*059, *η*_p_^2^ = .194, power = .480; invalid: *F*(1, 17) = 3.343, *p* = *.*085, *η*_p_^2^ = .164, power = .407]. These trends suggest that higher rewards speed up RTs when the exogenous cue is valid, whereas higher rewards slow RTs when the exogenous cue is invalid. Both effects diminish with lower rewards. No significant interaction between endogenous and exogenous cue validities was observed for any of the reward levels (all *F*s < 1).

**Fig. 3:**
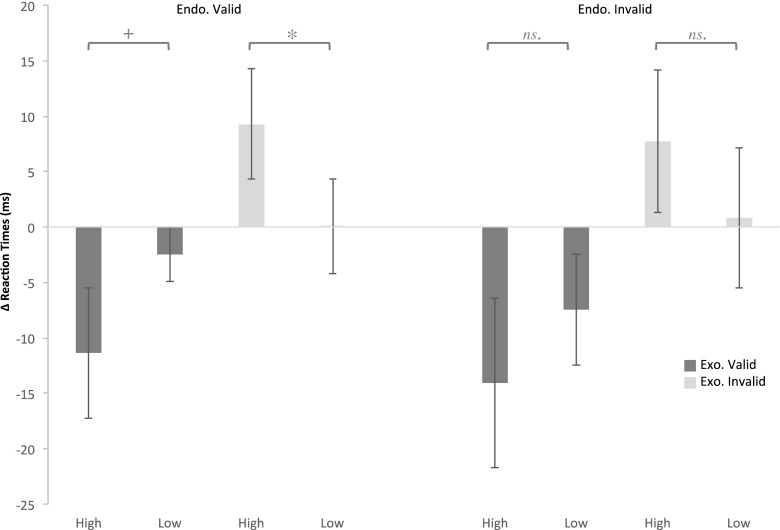
Changes in reaction times as a function of reward level, relative to no-reward trials. Error bars reflect the standard errors of the means

To obtain a better understanding of the costs and benefits associated with value-driven attentional capture, given a top-down attentional set, we investigated to what extent rewarded stimuli sped up and slowed down RTs relative to nonrewarded stimuli. Therefore, the average RTs obtained in the no-reward condition were subtracted from the RTs obtained in the high- and low-reward conditions, separately and for each cueing condition (see Fig. 3). A repeated measures ANOVA with Reward Difference (high, low) and Exogenous and Endogenous Cue Validities (valid, invalid) as within-subjects factors showed a main effect of exogenous cueing [*F*(1, 17) = 14.070, *p* = *.*002, *η*_p_^2^ = .453, power = .942]. Confirming the prior analyses, an Exogenous Cueing × Reward interaction was observed [*F*(1, 17) = 5.031, *p* = *.*039, *η*_p_^2^ = .228, power = .562]. No other interactions were observed in the data.

As can be observed in Fig. 3, there is a clear speeding up of RTs for rewarded, as compared to nonrewarded, trials when the exogenous cue was presented at the target’s location. Similarly, a slowing down can be observed when the exogenous cue was presented at the nontarget location. Planned paired-samples *t* tests between high (the difference between high and no reward) and low (the difference between low and no reward) differences, performed separately for each cueing condition, confirmed that speeding up and slowing down of RTs occurred when the *endogenous cue* was valid [a trend in the speed-up of RTs was observed for valid exogenously cued trials: *t*(17) = 1.967, *p* = *.*066], and a significant slowing down was observed for invalid exogenously cued trials [*t*(17) = 2.822, *p* = *.*012]. When the *endogenous cue* was invalid, the difference between high and low difference was not significant [exo. valid: *t*(17) = 1.061, *p* = *.*304; exo. invalid: *t*(17) = 1.045, *p* = *.*311; see Fig. 3]. Note that these results should be interpreted tentatively, because the main ANOVA did not show any significant interactions between endogenous cue validity and the other factors.

#### Accuracy

A repeated measures ANOVA on the percentages of correct responses, with reward level, endogenous cue validity, and exogenous cue validity as variables, revealed only a main effect of endogenous cue validity, indicating that participants responded more accurately to validly than to invalidly cued targets (92.6 % and 88.8 %, respectively) [*F*(1, 17) = 10.823, *p* = *.*004, *η*_p_^2^ = .389, power = .873]. No other main or interaction effects were observed. See Table 1 for all accuracy scores per condition.

### Control task

#### Reaction times

A repeated measures ANOVA was run with exogenous cue validity (valid, invalid) and endogenous cue validity (valid, invalid) as within-subjects variables. Since no reward was administered to the different colors, this factor was not included in the analysis. The results once again showed a clear endogenous cueing effect [*F*(1, 17) = 12.152, *p* = .003, *η*_p_^2^ = .417, power = .907]. Additionally, a strong exogenous cueing effect was observed [*F*(1, 17) = 9.601, *p* = .007, *η*_p_^2^ = .361, power = .831]. Both cueing effects showed faster RTs when the cue correctly indicated where the target would appear (endogenous: valid, 430 ms; invalid, 463 ms; exogenous: valid, 443 ms; invalid, 451 ms). Similar to the reward-cueing task, no interaction between the two factors was observed.

Although it appeared that participants were overall slightly faster in the reward-cueing task than in the control task (437 vs. 447 ms, respectively), an additional repeated measures ANOVA with task type (reward, control), reward level,^1^ exogenous cue validity, and endogenous cue validity as variables showed no main effect of task type [*F*(1, 17) = 2.179, *p* = .158, *η*_p_^2^ = .114, power = .286]. With regard to counterbalancing, ten participants first performed the reward task, whereas eight participants started with the control task. An ANOVA with Task Type, Reward Level, and both Cueing Validities as within-subjects factors and Task Order (reward first, control first) as a between-subjects factor showed a main effect of task order [*F*(1, 16) = 12.438, *p* = .003, *η*_p_^2^ = .437, power = .911], suggesting that when participants started out with the reward task, they remained fast throughout both experiments (445 and 439 ms, respectively, for the reward and control tasks), whereas participants who started out with the control task (457 ms) became faster when they switched to the reward task (428 ms). Crucially, order did not interact with any of the other variables (smallest *p* = .214).

We also addressed the question of whether the effects of exogenous attention were diminished for the no-reward trials in a reward context, as compared to the no-reward trials in a no-reward context. In order to investigate this, we ran a repeated measures ANOVA with context (reward vs. control), exogenous cue validity (valid, invalid), and endogenous cue validity (valid, invalid) as variables, on no-reward trials only. That is, only RTs from the no-reward condition in the reward task were used, as well as all RTs from the control task, because there was never a reward in the latter task. We found no influence of context [*F*(1, 17) = 1.249, *p* = .279, *η*_p_^2^ = .068, power = .184], nor did context interact with any of the other factors (*p*s > .244).

#### Accuracy

An ANOVA on accuracy scores with reward level and both cue validities as variables showed a main effect of endogenous cueing [*F*(1, 17) = 13.062, *p* = *.*002, *η*_p_^2^ = .435, power = .926], with participants being more accurate for valid than for invalid endogenous cues (92.1 % and 86.2 % correct, respectively). No other significant effects were observed.

## Discussion

In the present study, we investigated to what extent differential reward values influenced attentional capture while a top-down set was activated. To this end, we had participants perform a Posner cueing task. The results showed strong endogenous and exogenous cueing effects, indicating that participants were faster when attention was deployed to a valid target location. In addition, a highly significant interaction between reward level and exogenous cue validity was observed, showing that stimuli associated with higher reward values captured more attention, resulting in sped-up RTs when reward-associated cues signaled the correct target location. By contrast, when the reward-associated cue signaled the distractor location, slowed RTs were observed. This is in line with recent findings indicating that the value attached to specific stimuli captures more attention, independent of the physical features of such stimuli (Anderson, Laurent, & Yantis, 2011; Anderson, 2013; Hickey, Chelazzi, & Theeuwes, 2010, 2011; Le Pelley et al., 2015).

In the control task, in which no reward was distributed, classic attentional effects were observed showing faster RTs when participants were endogenously cued with the location of the target than when no such information was available (Posner, 1980; Theeuwes & van der Burg, 2007). Furthermore, faster RTs were observed when a salient exogenous cue was presented at the location of the target than when it was presented at the nontarget location (see, e.g., Theeuwes, 1992; for an overview of endogenous and exogenous attentional effects on perception, see Theeuwes, 2010). Importantly, both types of cueing appeared to operate independently of each other and seemingly at the same time—a finding that has also been observed in nonreward studies (Berger & Henik, 2000; Chica, Botta, Lupiáñez, & Bartolomeo, 2012).

One of the strengths of the present paradigm is that reward value exerted an effect on attentional capture in the absence of a training phase in which the rewarded target was repeatedly selected (Anderson, 2013; Anderson, Laurent, & Yantis, 2011). A training phase in which the rewarded feature was directly associated and co-selected with the target could have explained the present results in terms of a selection bias for the rewarded feature, rather than of value-driven attentional capture. In the present paradigm, this direct association between the reward feature and the target was absent. As such, these findings show that associative learning occurs rapidly, and therefore they clearly show that associative-learning processes automatically influence attentional capture by increasing signal value, independent of selection history (Anderson, 2013). Importantly, motivational effects are not likely to have influenced the present results. First, reward value was related to the exogenous cue that was presented simultaneously with the target, rendering motivational effects unlikely to occur. Second, there was no effect of reward on the overall RTs in the two tasks. That is, if the reward task had made participants more motivated to perform, faster RTs would have been expected. This was not observed, and therefore effectively rules out any influence of motivational or endogenous attention effects, further evidenced by the absence of an interaction between endogenous attention and reward.

Interestingly, the attentional capture effect was no longer observed for the no-reward trials in the reward-cueing task. The absence of this effect may suggest that the attentional capture observed in the high- and low-reward conditions was purely value driven. However, the control task showed a strong saliency-driven attentional capture effect in the absence of any reward. The discrepancy in attentional capture for nonrewarded exogenous cueing in a rewarded or a nonrewarded context may therefore relate to the present paradigm: Due to the use of a relatively sparse display, the value-driven attentional set was prioritized over saliency-driven attentional set. This allowed rewarded stimuli to exert a larger influence on attentional guidance than did salience, even when reward was absent. That is, value-driven attentional capture in this particular paradigm may abolish some of the saliency-driven attentional capture. On the contrary, when there is no value-driven attentional set, even relatively weak salient stimuli will capture attention.

Although the present study showed a speeding up and a slowing down for valid and invalid exogenous cues, respectively, it is important to note that the relative speeding up and slowing down for valid and invalid exogenous cues can only be perceived in relationship to each other. It is not possible to make any specific claims concerning how reward-associated cues operate in relationship to a baseline, because the present experiment did not have a baseline condition (e.g., a nonreward/noncolored cue). Therefore, we cannot make a strong claim as to the extent to which reward-associated cues lead to a benefit at the target location, as compared to the costs at a nontarget location.

Furthermore, the absence of an interaction between endogenous cue validity and reward level showed that the reward effects were not stronger or weaker when the endogenous cue predicted the target location correctly, as compared to when it did not. Since the top-down cue focuses attention before the target is presented, initially attention is equally focused at valid and invalid locations. Classic studies on endogenous attention (e.g., Theeuwes, 1991) often manipulated the extent to which attention was focused by using valid and invalid (focused) cues as well as neutral cues that provided no information about a target’s upcoming location. Because we did not use neutral cues in the present study, we could only compare the two focused conditions, leading to a main effect of endogenous cueing, but not to an interaction with reward value.

One of the possible pitfalls of the present study is that eye movements were not measured during the experiment. Given that participants could earn a reward based on accurate and fast performance, they might have strategically initiated eye movements on the basis of the highly valid endogenous cues. This would lead to the situation in which, on valid endogenous trials, the exogenous cue was foveally attended, whereas on invalid endogenous trials, the exogenous cue was presented periphally. These differences in attentional allocation due to eye movements could theoretically lead to the finding that participants were slower on invalid than on valid exogenous trials, as was the case in the present study.

However, there are a number of reasons why it is unlikely that eye movements can explain our data. First, if participants would have made eye movements to the cued location, they simply would not be able to respond to the target when it happened to appear at the uncued location. Because the target was only presented for 200 ms, participants would have been too late to perceive the target when they had to make a saccade to the uncued location. Because it was impossible to identify the target when presented at an uncued location while fixating the cued location, one would have expected an accuracy score approaching chance level (but see Yeshurun & Rashal, 2010, for an alternative explanation). As is clear from the present data, accuracy scores were found to be approximately 89 % correct. Second, previous (classic) research has shown that participants can perform these tasks without making eye movements when they are properly instructed (as was the case in the present study; see, e.g., Müller & Rabbit, 1989, and Yantis & Jonides, 1990). Third, if participants had made eye movements to the expected target location based on an invalid endogenous cue, and subsequently had to make an eye movement toward the actual target location, it would follow that the RT difference between valid and invalid trials would be roughly 200–250 ms, reflecting the time it would take to make an eye movement. However, as can be seen in Table 1, the differences in RTs for valid and invalid endogenously cued trials ranged between 20 and 30 ms, a range that is typical for this type of experiment. Together, it seems highly unlikely that participants made eye movements, and as such, we believe that eye movements in the cued direction cannot explain our findings.

To summarize, the present results support the notion that associative reward learning exerts a strong influence on attentional guidance. The results also show that contingencies between stimulus features and reward value can be learned rapidly, which, in turn, increases the relative importance of rewarded features on an attentional priority map (Awh, Belopolsky, & Theeuwes, 2012). This suggests that, under certain conditions, value-driven attention can exert a stronger effect on perception than does saliency-driven attentional guidance, and that value-driven attentional capture operates independent of endogenous attention.

## Footnotes

1 Please note that “reward level” was a hypothetical construct in the control task. Although participants did not obtain rewards in the control task, we added this factor to create a full factorial design. Similar color–reward schemes were used in both tasks. For example, any participant that had a red–high reward association in the reward task would have a green–high reward association in the control task. Interpreting this dataset beyond the main effect of task type is not advised.
